# Risk factors for the incidence of apoplexy in pituitary adenoma: a single-center study from southwestern China

**DOI:** 10.1186/s41016-020-00202-4

**Published:** 2020-07-07

**Authors:** Yao Li, Yuan Qian, Yisheng Qiao, Xiaoxiang Chen, Jiaotian Xu, Chao Zhang, Wei Wang, Junjun Li, Xingli Deng

**Affiliations:** 1 Department of Neurological Surgery, 1st Affiliated Hospital of Kunming Medical University, Kunming, China; 2 Yunnan Key Laboratory of Laboratory Medicine, Yunnan Engineering Technology Center of Digestive disease, 1st Affiliated Hospital of Kunming Medical University, Kunming, China; 3 Department of Medical Genetics and Prenatal Diagnosis, Kunming Maternal and Child Health Hospital, Kunming, China

**Keywords:** Pituitary adenoma, Pituitary apoplexy, Risk factors

## Abstract

**Background:**

Although the incidence and clinical manifestations of pituitary apoplexy were reported by a few researches, the results are not consistent. This study aimed to explore the risk factors associated with an incidence of apoplexy in pituitary adenomas.

**Methods:**

The clinical information of 843 patients with pituitary adenoma from the Department of Neurological Surgery, 1st Affiliated Hospital of Kunming Medical University, was reviewed. The incidence, clinical manifestation, and potential risk factors for pituitary apoplexy were analyzed by a case-control study.

**Results:**

In total, 121 patients (14.4%) with macroadenoma were suffered from pituitary apoplexy. Headache, vomiting, and visual impairment are the top 3 symptoms for the pituitary apoplexy.

Logistic regression results showed that the hypertension(hypertension vs non-hypertension OR = 2.765, 95%CI:1.41~5.416), tumor type (negative staining vs. positive staining, OR = 1.501, 95%CI:1.248~5.235), and tumor size (diameter > 2 cm vs. diameter ≤ 2 cm, OR = 3.952, 95%CI:2.211~7.053) are independent factors associated with pituitary apoplexy.

**Conclusion:**

Our results indicate that the risk factors for the incidence of pituitary apoplexy depend mainly on properties of the tumor itself (tumor size and pathologic type) and the blood pressure of patients.

## Background

In 1950, the term “pituitary apoplexy” was first formally used to describe a fatal case of pituitary tumor-associated hemorrhage or *infarction* [1]. Patients suffering from pituitary apoplexy usually complain about an unexpected severe headache, sometimes visual deterioration [2, 3].

Acute apoplexy is unpredictable and complicated [4]. Two distinct extreme manifestations of acute apoplexy are observed in health centers: patients suspected with pituitary apoplexy may recover immediately without neurological and endocrinological deficiency or deteriorate intensely owing to mass effects and the secondary subarachnoid hemorrhage (SAH) [5]. Moreover, in some cases, the tumor may be completely controlled after apoplexy while the remnants may continue to grow in other cases [6, 7].

Doctors including emergency physicians, ophthalmologists, endocrinologists, and neurosurgeons are facing critical issues raised by the management of pituitary apoplexy. However, rare studies about pituitary apoplexy have been reported, and there is few evidence-based strategies to manage these patients [8]. In the past, pituitary apoplexy was treated as an emergence situation and immediate surgical intervention was preferable. Nevertheless, conservative treatment is also desirable nowadays [9]. Thus, the optimal management of pituitary apoplexy remains a controversy.

Identifying risk factors of pituitary apoplexy would do a favor for the management of pituitary apoplexy. To explore the risk factors associated with the incidence of pituitary apoplexy, we reviewed and analyzed the clinical information of 843 patients with pituitary adenomas (PA) from the Department of Neurological Surgery, 1st Affiliated Hospital of Kunming Medical University.

## Methods

Patients attended at the Department of Neurological Surgery, 1st Affiliated Hospital of Kunming Medical University, and diagnosed with PA from 2013 to 2017 were included in our study. All patients had operations either trans-sphenoidal microsurgery or craniotomy. Eight hundred forty-three patients aged from 15 to 82 (mean, 45.0 ± 13.4 years) were recruited. The research had achieved authorization from the Ethics Committee of the First Affiliated Hospital of Kunming Medical University.

### Data collection

Data on clinical features including sex, age, main complaint, clinical manifestation, results of imaging examination, endocrine function tests, pathological results, and medical history were obtained from the medical record database.

### Cases and controls

The factors influencing the incidence of pituitary apoplexy were studied by a matched case-control study. Patients with pituitary apoplexy (case group) were contrasted with surplus PA patients without pituitary apoplexy (control group). Patients of the control group were selected by a computer-generated random selection scheme. Control patients were matched to case patients by age (cases: controls = 1:2).

### Diagnostic criteria

Patients with two or three of the following standards were diagnosed with pituitary apoplexy:

1. Obvious symptoms of pituitary apoplexy (for instance, sudden headache, or vomiting, or visual impairment);
2. Radiologic signs of hemorrhage;
3. Intraoperative or pathological found hemorrhage;

Hypertension is defined as taking hypertensive drugs or systolic blood pressure (≥ 140 mmHg) or diastolic blood pressure (≥ 90 mmHg) [10]. Diabetes mellitus (DM) was diagnosed according to WHO diagnostic criteria in 1999 [11].

### Imaging examination

All patients received a computed tomography (CT) and/or magnetic resonance imaging (MRI) scan. Three-dimensional imaging provided by MRI shows the location and the degree of hemorrhage.

### Pathology examination

Standard immunohistochemical staining was used to determine tumor types at the Department of Pathology, The First Affiliated Hospital of Kunming Medical University.

### Statistical methods

All quantity datas were analyzed by SPSS software (version 24.0). The main method is to compare the frequency distribution of each group by general statistical description, Pearson’s chi-squared test, and Pearson’s statistic with a continuity correction. In order to evaluate the risk factors of pituitary apoplexy, logistic regression analysis was carried out to adjust the confounding factors in the whole group of patients; Calculation sheet variables and the adjusted odds ratio (OR) and corresponding 95% confidence interval (CI). Two-tailed *P* values less than 0.05 were considered statistically significant.

## Results

**Fig. 1:**
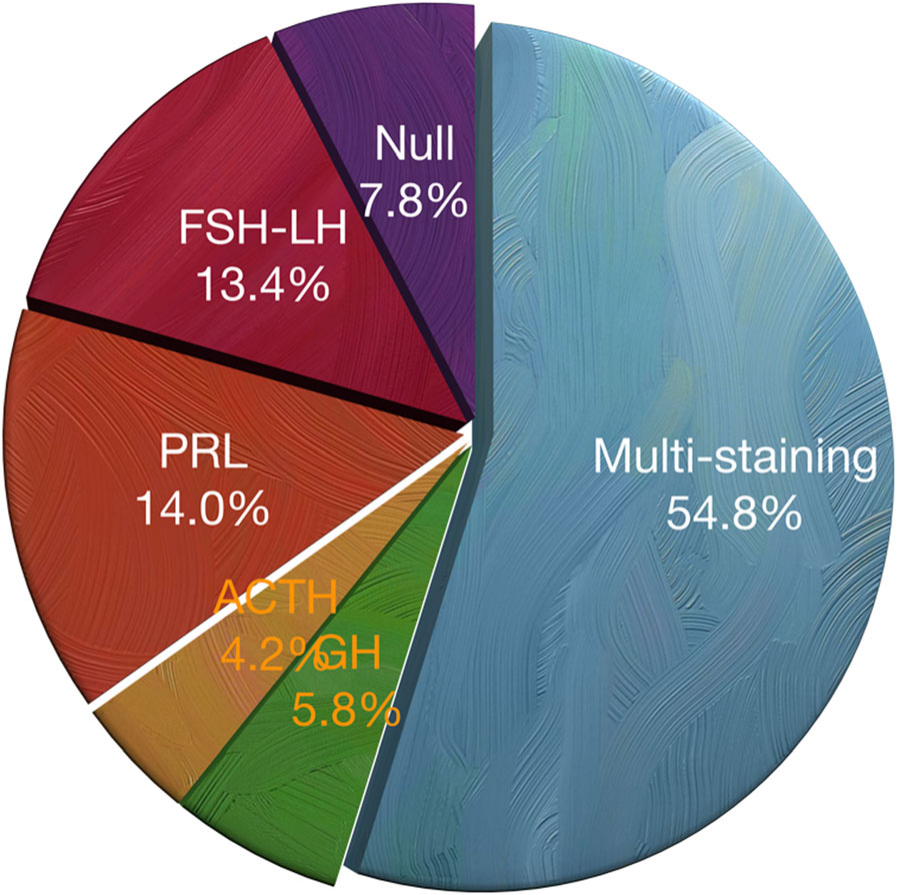
The subtype of tumors based upon immunohistochemical results. PRL, prolactin; GH, growth hormone; ACTH, adrenocorticotropic hormone; FSH-LH, gonadotropin hormone. Multiple stain, multi-hormone

Eight hundred forty-three patients (mean age: 45.0 ± 13.4 years) with PA were included in this study. Table 1 shows the clinical characteristics of these patients, and the tumor subtypes based on pathological results are shown in Fig. 1.

**Table 1:** Characteristics of patients with pituitary tumors (*n* = 843, January 2008–December 2018)

Characteristics	*n*	%
Age		
≤ 35	207	24.6
36~45	228	27.0
46~55	224	26.6
≥ 56	184	21.8
Gender		
Female	401	47.6
Male	442	52.4
Tumor size		
*d* ≤ 2 cm	267	31.7
*d* > 2 cm	576	68.3
Diabetes mellitus	42	5
Hypertension	125	14.8
Pituitary apoplexy	121	14.4

### Incidence of pituitary apoplexy

One hundred twenty-one (14.4%) PA patients, with a mean age of 43.5 ± 14.3 years, suffered from pituitary apoplexy, As showed in Table 2, there was no significant difference in the incidence of pituitary apoplexy between different ages (*P* = 0.993, Table 2). And pituitary apoplexy occurred approximately equal between men (7.82%) and women (6.52%). In addition, there was no significant correlation between tumor size and pituitary apoplexy in unmatched patients(*P* = 0.276).

**Table 2:** The incidence of pituitary apoplexy

Characteristics	Apoplexy(*n* = 121)	%	*P*
Age			
≤ 35	33	3.91	0.993^*^
36~45	31	3.67	
46~55	35	4.15	
≥ 56	22	2.61	
Gender			
Female	66	7.82	0.228^*^
Male	55	6.52	
Tumor size			
*d* ≤ 2 cm	38	4.50	0.276
*d* > 2 cm	83	9.84	
Diabetes mellitus	5	0.59	0.563^*^
Hypertension	22	2.61	0.006^*^
Pathological staining			
Null	66	7.8	NA
FSH-LH	113	13.4	
PRL	118	14.0	
ACTH	35	4.2	
GH	49	5.8	
TSH	0	0	
Multi-staining	462	54.8	

*NA* Not applicable, *PRL* Prolactin, *GH* Growth hormone, *LH* Luteinizing hormone, *ACTH* Adrenocorticotropic hormone, *TSH* Thyroid-stimulating hormone, *FSH* Follicle-stimulating hormone, *PA* Clinical pituitary apoplexy

*Pearson’s chi-squared test

### Clinical presentation

Table 3 illustrated the comparison of clinical presentation between pituitary apoplexy cases (*n* = 121) and controls (*n* = 242). The top 3 common symptoms in pituitary apoplexy were headache, visual deterioration, and visual field defect. These three symptoms were also the top 3 in patients without pituitary apoplexy.

**Table 3:** Comparison of clinical presentation between pituitary apoplexy cases (*n* = 121) and controls (*n* = 242)

Symptom/sign	Pituitary apoplexy (*n* = 121)		control (*n* = 242)		*P*
	*n*	%	*n*	%	
Headache	87	71.9	130	53.7	0.001^*^
Vomiting	23	19.0	14	5.80	0.000^*^
Fever	3	2.5	5	2.10	0.800^*^
Visual impairment	80	66.1	155	64.0	0.698^*^
Diplopia	2	1.7	7	2.90	0.474^*^
Ablepsia	12	10.0	11	4.50	0.045^*^
Visual field defect	51	50.4	124	51.20	0.882^*^
Acromegaly	5	4.1	18	7.40	0.223^*^
Cushingoid appearance	0	0	4	1.7	0.356^†^
Sexual hypoactivity^b^	0	0	3	1.2	0.463^†^
Hypopituitarism	20	16.5	28	11.6	0.189^*^
Hypothyroidism	32	26.45	31	12.81	0.001^*^
Amenorrhea^a^	16	15.84	72	12.57	0.542^*^
Altered menstrual cycle^a^	14	11.57	26	10.74	0.406^*^
Galactorrhoea^a^	19	15.70	23	9.50	0.026^*^
Gynecomastia^b^	0	0	0	0	–

*Pearson’s chi-squared test

^a^These calculations include only women

^†^Pearson’s statistic with a continuity correction

^b^These calculations include only men

### Risk factors for pituitary apoplexy

The results of multiple logistic regression analysis indicated that age and sex were not associated with the incidence of pituitary apoplexy (*P* > 0.05). However, tumor size and pathologic type were confirmed to be associated with pituitary apoplexy occurrence. In comparison with positively pathological staining tumors, negative pathological staining ones were associated with the occurrence of pituitary apoplexy (OR:1.501, CI:1.248~5.235, *P* < 0.05). In addition, comparing with the tumors with a diameter equal/less than 2 cm, the tumors with a diameter of more than 2 cm were associated with pituitary apoplexy incidence (OR:3952, CI:2.211~7.053, *P* < 0.05). Furthermore, a history of hypertension was also significantly associated with the incidence of pituitary apoplexy (Table 4).

**Table 4:** Risk factors for incidence of PA in pituitary adenoma patients

Factors	Univariate OR(95%Cl)	*P*	Adjusted OR(95%Cl)	*P*
Age (years)				
≤ 35^a^	1			
36~45	1.068 (0.554~2.059)	0.844	0.871 (0.463~1.640)	0.668
46~55	0.929 (0.558~2.113)	0.809	0.744 (0.395~1.402)	0.360
≥ 56	0.775 (0.574~2.106)	0.755	0.530 (0.253~1.109)	0.092
Gender				
Female^a^	1		1	
Male	1.312 (0.844~2.040)	0.228	0.849 (0.553~1.340)	0.455
Tumor size				
*d* ≤ 2 cm^a^	1		1	
*d* > 2 cm	3.676 (2.0821~6.491)	0.000	3.952 (2.211~7.053)	0.000
Diabetes mellitus	0.759 (0.315~1.832)	0.540	0.774 (0.315~1.903)	0.577
Hypertension	2.467 (1.287~4.726)	0.006	2.765 (1.411~5.416)	0.007
Pathological staining				
Positive staining^a,b^	1		1	
Negative staining^c^	0.382 (0.192~0.759)	0.006	1.501 (1.248~5.235)	0.010

^a^Reference

^b^Including PRL, FSH, GH, ACTH, TSH, and multiple staining

^c^Null cell adenoma

## Discussion

The classical term “pituitary apoplexy” [1] refers to a clinical syndrome characterized by abrupt onset of headache accompanied by neurologic or endocrinologic deterioration due to a sudden expansion of a mass within the sella turcica as a result of hemorrhage, infarction, or necrosis within a pituitary tumor and adjacent pituitary gland [2].

The incidence of pituitary apoplexy varies from 0.6 to 22% with different diagnostic criteria. Our study shows that 14.4% PA patients suffered from pituitary apoplexy, which is a little higher than some previous studies and may be related to the disparity in diagnosis. The diagnosis of pituitary apoplexy mainly bases on clinical presentations and imaging results. In this study, however, pituitary apoplexy was diagnosed on the basis of clinical signs couple with imaging, intra-operative, or histopathological findings [12–16].

The clinical presentation of pituitary apoplexy could be acute or subacute, depending on the amount of hemorrhage and its incidence speed. Usually, headache is the most frequent and earliest symptom of pituitary apoplexy, which is caused by the stimulation and stretching of the hypophyseal capsule or/and hemorrhage into subarachnoid space [17, 18]. Our study confirmed that 71.9% pituitary apoplexy patients present with a headache, which is consistent with previous studies. Besides, our study showed visual impairment was the second most common symptom of pituitary apoplexy, which is consistent with the previous report [19].

The risk factors for pituitary apoplexy are inconsistent between studies. The reported risk factors can be sort into categories: (1) reduced blood flow for the tumor, such as large tumor size; (2) acute increase in hypophyseal blood flow, including hypertension, diabetes, trauma, and increased intracranial pressure; (3) hormonal stimulation of the pituitary gland and tumor, for instance, endocrine stimulation tests, pregnancy, and exogenous estrogen therapy; (4) anticoagulated state, for example, anticoagulation, thrombolytic, and antiplatelet therapy [2, 16, 20–22]. Zhu et al. analyzed the incidence of pituitary apoplexy in 2021 PA cases and recognized male sex, non-function adenomas along with macroadenomas as risk factors for pituitary apoplexy [23].

Similar to the results of previous studies [24], our results show that the incidence of pituitary apoplexy in males(6.52%)is similar to females and is also very close between different age groups (Table 2), which means that sex and age are not independent risk factors for pituitary apoplexy.

Consistent with findings of previous studies [16, 20, 21, 23], the incidence of pituitary apoplexy is correlated with tumor size in our results. Pituitary apoplexy occurred near 4 times more often in large-sized tumors (diameter more than 2 cm) than small ones (diameter equal/less than 2 cm) (*P* = 0.000 < 0.05 OR:3.952, 95%CI:2.211~7.053). Therefore, we believe that tumor size is a risk factor for pituitary apoplexy.

It remains controversial whether the subtypes of PAs are a risk factor for the development of apoplexy. Previous studies reported that up to 45% of pituitary apoplexy were developed from non-function adenomas [2, 25]. However, a study demonstrated that secreting pituitary adenomas had a higher incidence of apoplexy [26]. Similar to results by Zhu’s works [23], our study showed that the incidence of pituitary apoplexy is more common in pathological negative staining PA patients. The incidence of pituitary apoplexy in pathological negative staining adenoma is approximately 1.248~5.235 times higher than pathological positive staining adenomas, which means that the pathological negative staining of a tumor is an independent risk factor for pituitary apoplexy.

As it is thought, diabetes mellitus(DM)and arterial hypertension could affect the perfusion of microvasculature in the pituitary gland and pituitary adenoma [2, 27–29]. In our study, 2.61% and 0.59% of patients with pituitary apoplexy suffered from hypertension and DM, respectively. We found that hypertension is an independent risk factor for pituitary apoplexy through multivariate regression analysis with an OR of 2.765 (95%CI:1.411–2.231). However, it has not been proved that DM is associated with pituitary apoplexy.

## Conclusions

We conclude that the incidence of pituitary apoplexy is much more frequent than previously assumed. The main risk factors for pituitary apoplexy are properties of the tumor (tumor size and pathologic type) and the blood pressure of patients, which may help the earlier diagnosis of pituitary apoplexy in patients with PAs.

## Data Availability

Please contact the authors for data requests.

## Abbreviations

CI: Confidence interval
CT: Computed tomography
DM: Diabetes mellitus
MRI: Magnetic resonance imaging
OR: Odds ratio
SPSS: Statistical Package for Social Sciences
